# Preparation of High-Porosity B-TiO_2_/C_3_N_4_ Composite Materials: Adsorption–Degradation Capacity and Photo-Regeneration Properties

**DOI:** 10.3390/ijerph19148683

**Published:** 2022-07-17

**Authors:** Xiang Guo, Lei Rao, Zhenyu Shi

**Affiliations:** 1 College of Environment, Hohai University, Nanjing 210098, China; gx2015@hhu.edu.cn (X.G.); szy@hhu.edu.cn (Z.S.); 2 College of Mechanics and Materials, Hohai University, Nanjing 211100, China

**Keywords:** adsorption, degradation, B_2_O_3_, TiO_2_/C_3_N_4_, molten

## Abstract

Adsorption can quickly remove pollutants in water, while photocatalysis can effectively decompose organic matter. B-TiO_2_/g-C_3_N_4_ ternary composite photocatalytic materials were prepared by molten method, and their adsorption–degradation capability under visible light conditions was discussed. The morphology of the B-TiO_2_/g-C_3_N_4_ materials was inspected by SEM, TEM, BET, and EDS, and the results showed that close interfacial connections between TiO_2_ and g-C_3_N_4_, which are favorable for charge transfer between these two semiconductors, formed heterojunctions with suitable band structure which was contributed by the molten B_2_O_3_. Meanwhile, the molten B_2_O_3_ effectively increased the specific surface area of TiO_2_/C_3_N_4_ materials, thereby increasing the active sites and reducing the recombination of photogenerated electron–hole pairs and improving the photocatalytic degradation abilities of TiO_2_ and g-C_3_N_4_. Elsewhere, the crystal structure analysis (XRD, XPS, FTIR) results indicated that the polar -B=O bond formed a new structure with TiO_2_ and g-C_3_N_4_, which is not only beneficial for inhibiting the recombination of electron holes but also improving the photocatalytic activity. By removal experiment, the adsorption and degradation performances of B-TiO_2_/g-C_3_N_4_ composite material were found to be 8.5 times and 3.4 times higher than that of g-C_3_N_4_. Above all, this study prepared a material for removing water pollutants with high efficiency and provides theoretical support and experimental basis for the research on the synergistic removal of pollutants by adsorption and photocatalysis.

## 1. Introduction

Photocatalysis is a technology that can use semiconductor materials to remove pollutants from the environment under different lighting conditions [1,2,3]. Most materials require ultraviolet conditions to produce redox effects. The visible part of sunlight irradiate that reaches the water surface only occupies 45%, while the ultraviolet part occupies less than 4% [4]. At the same time, water absorbs and reflects sunlight in different wavelengths, which seriously limits the removal efficiency of photocatalytic materials in a water environment [5,6,7].

g-C_3_N_4_ is a good semiconductor material with application potential that is metal free and has visible light responsiveness [8,9,10]. However, researchers found that g-C_3_N_4_ still has many disadvantages such as low utilization of visible light, poor photoelectric conversion efficiency, and low specific surface area [11]. Since Serpone et al. [12] first reported that a solid–solid heterojunction interface with good contact can be constructed from different coupled semiconductor materials to promote electron transfer between particles, more and more researchers have paid attention to different semiconductor materials. Among the many photocatalytic semiconductor materials, TiO_2_ is widely used in sewage treatment [13], photocatalytic synthesis [14], and self-cleaning [15]. Due to its easy availability, low cost, stable chemical properties, corrosion resistance, non-toxicity, and strong oxidizing properties [16], its air purification [17] and antibacterial properties [18] have been extensively studied. Therefore, using the advantages of TiO_2_ material properties to composite it with g-C_3_N_4_ to become a more competitive material has attracted the attention of more and more researchers [19,20,21].

In the treatment of water environment pollution, the adsorption performance and visible light response ability of photocatalytic materials are two important factors that determine whether the photocatalytic technology can be effectively promoted [22,23]. The high-efficiency adsorption and enrichment ability of the material reduce the concentration of pollutants in water and provide a high-concentration contact environment that is conducive to the photocatalytic reaction for the material [24,25]. The melting characteristics of B_2_O_3_-modified g-C_3_N_4_ are effective for improving its specific surface area and adsorption performance, but its response to visible light is limited, which affects the visible light photocatalytic activity [26].

Effectively improving the adsorption performance and visible light catalytic activity of materials at the same time is a key issue for researchers. Functional coupling through different materials is an idea to solve this problem. Here, TiO_2_ and g-C_3_N_4_ were formed into a composite heterostructure to improve the photocatalytic oxidation ability of the material. Then, the melting characteristics of B_2_O_3_ during the heating process were used as a “reaction environment regulator”, and TiO_2_/C_3_N_4_ was co-calcined to prepare a composite material. The results of the experiment showed that B element doped the composite-modified materials, and, finally, the adsorption behavior and visible light catalytic degradation ability of B-TiO_2_/C_3_N_4_ were analyzed by using MB and RhB organic pollutants. This method effectively increased the specific surface area of the material, increased the conjugated system, improved the adsorption capacity, and effectively improved the visible light catalytic effect of the material. This provides a technical idea for the promotion and application of a low-cost preparation of an efficient adsorption–degradation photocatalytic composite material.

## 2. Materials and Methods

### 2.1. Sample Preparation

(1) TiO_2_: 17 mL of butyl titanate (Aladdin, 99% pure) was placed into 55 mL of ethanol solution (Aladdin, 99.7% pure), then 4.5 mL of glacial acetic acid (Aladdin, 99% pure) was added. After mixing using a magnetic stirrer, the solution was recorded as A. We measured 27.6 mL of ethanol, added 0.9 mL of distilled water, and adjusted the pH to 4 with nitric acid (Sinopharm, 65−68% pure), and this solution was recorded as B after thorough mixing. The solutions A and B were stirred for 30 min respectively, the B solution was added dropwise to the A solution at a rate of 10 drops per minute, and the final obtained mixed solution was TiO_2_ sol. Then, the TiO_2_ sol was stirred at room temperature and placed in an oven to dry after gelation. Then, the dried xerogel was ground into powder and placed in a crucible with a lid to heat at a rate of 5 °C/min until 550 °C and was maintained at this temperature for 2 h. After the temperature in the muffle furnace (XS2−10, Li Chen, China) dropped to room temperature, the calcined material was ground into powder, and the obtained material was denoted as TiO_2_.
(2) g-C_3_N_4_: 10 g melamine (Aladdin, 99% pure) was placed into a 50 mL crucible and put into muffle furnace, and the heating rate was set to 5 °C/min until it reached the reaction temperature of 550 °C. Then, the reaction temperature was maintained for 2 h. After the material cooled to room temperature, we ground the calcined solid, and the obtained yellow powder was g-C_3_N_4_.
(3) TiO_2_/C_3_N_4_ composite material: 1 g of melamine and 10 mL of TiO_2_ sol were weighed in a beaker, stirred, and mixed thoroughly and then the mixture was placed into an oven for drying after gelation. The dried composite precursor was placed in a crucible with a lid and calcined at 550 °C for 2 h. The obtained material was ground into powder after cooling to room temperature to obtain TiO_2_/C_3_N_4_ composite material.
(4) B-C_3_N_4_: The B_2_O_3_ (Aladdin, 99.9% pure) and g-C_3_N_4_ were mixed and ground evenly. Then, the composite powder was placed into a ceramic crucible. After that, the samples were heated to 550 °C at a heating rate of 5 °C/min and maintained for 2 h. When the furnace was cooled to room temperature, the powders were washed with water and ethanol several times. For convenience of description, the composite material was abbreviated as B-C_3_N_4_.
(5) B-TiO_2_/C_3_N_4_: The TiO_2_/C_3_N_4_ material and B_2_O_3_ were mixed and stirred in distilled water at a mass ratio of 1:1. After grinding evenly, we put it into an atmosphere furnace at 550 °C for 2 h. Then, the material was soaked and washed three times with ethanol and distilled water, respectively. The sample was denoted as B-TiO_2_/C_3_N_4_. For comparison, another ternary composite material, B2-C_3_N_4_/TiO_2_, was prepared: 1 g of B-C_3_N_4_ and 10 mL of TiO_2_ sol were weighed with the same method.

### 2.2. Characterization

The structural analysis of the samples was carried out by X-ray diffraction (XRD, Rigaku Ultima Ⅲ, Tokyo, Japan) and was recorded in the 2θ range of 5–80° with a scan rate of 0.02°/0.4 s using a Bruker AXSD8 system (Bruker, Billerica, MA, USA) equipped with a Cu Kα radiation source (λ = 0.15406 Å, in which the X-ray tube was operated at 40 kV and 40 mA). UV–Vis diffuse reflectance spectra (DRS) were obtained on a UV–visible (UV–Vis) spectrophotometer (PerkinElmer, Waltham, MA, USA) with BaSO_4_ as the reference. The sample morphology of the different composite materials was examined using a scanning electron microscope (SEM, Hitachi, Tokyo, Japan) and a transmission electron microscope (TEM, JEM-2100, JEOL, Tokyo, Japan). X-ray photoelectron spectroscopy (XPS) measurements were performed on a Thermo Scientific ESCALAB 250 instrument (Thermo Fisher Scientific, Waltham, MA, USA) with an Al Kα source. Low-temperature N_2_ adsorption/desorption measurements (Brunauer–Emmett–Teller (BET) method) were carried out using a Micromeritics ASAP 2020 system (Micromeritics, Norcross, GA, USA) at −196 °C following degassing of all samples at 120 °C for 2 h.

### 2.3. Adsorption and Photocatalytic Degradation of Organic Pollution

In order to evaluate whether composite materials have a better effect on the removal of pollutants, TiO_2_, g-C_3_N_4_, B-TiO_2_, B-C_3_N_4_, TiO_2_/C_3_N_4_, B-TiO_2_/C_3_N_4_, and B2-C_3_N_4_/TiO_2_ were weighed in the removal experimental. A 30 mg amount of each of material was added to the MB solution with a volume of 100 mL and a concentration of 20 mg/L. Under the condition of magnetic stirring, the photocatalytic efficiency was measured after 30 min of dark reaction adsorption experiment. A 1.5 mL amount of solution was taken out for measurement each time, and the removal rate of pollutants was determined by the following formula:

(1) $$R_{t}=\frac{C_{0}-C_{t}}{C_{0}}\times 100\%$$

where *R_t_* is the removal rate at time *t* after commencing the adsorption and photocatalytic degradation process, and *C*_0_ and *C_t_* are initial concentration and concentration at time *t*, respectively.

(2) $$Qe=\frac{C_{0}-C_{e}}{m}V$$

where *Qe* is the adsorbed quantity of samples at the equilibrium moment of adsorption and desorption. *C*_0_, *C_e_*, *V*, and *m* are the initial concentration, concentration at time *t*, initial volume of MB, and quantity of adsorbent, respectively.

### 2.4. Kinetics and Adsorption Isotherm Model

The adsorption behaviors were fitted to the materials by pseudo-first-order kinetic and pseudo-second-order kinetic models, respectively. Two kinetic linear models are shown below [27]:

Pseudo-first-order kinetic model:

(3) $$ln\left(Q_{e}-Q_{t}\right)=lnQ_{e}-k_{1}t$$

Pseudo-second-order kinetic model:

(4) $$\frac{t}{Q_{t}}=\frac{1}{k_{2}Q_{e}^{2}}+\frac{t}{Q_{e}}$$

where *Q_t_* and *Q_e_* in the formula are the adsorption amount of the sample at time *t* and equilibrium, respectively. *k*_1_ and *k*_2_ are pseudo-first-order kinetic constants and pseudo-second-order kinetic constants, respectively.

Langmuir and Freundlich are the two most common adsorption isotherm models. The Langmuir model assumes that the surface of the adsorbate is uniform and the adsorption capacity is the same everywhere, and it only occurs on the outer surface of the adsorbent; this is monolayer adsorption. The Freundlich adsorption equation can be applied to both monolayer adsorption and adsorption behavior on uneven surfaces. Freundlich, as an empirical adsorption isotherm for non-uniform surfaces, is more applicable to low-concentration adsorption and can interpret experimental results over a wider concentration range. Therefore, in this experiment, the experimental results are fitted by these two adsorption isotherm models, and the formulas are as follows [28]:

Langmuir:

(5) $$\frac{C_{e}}{Q_{e}}=\frac{1}{Q_{m}K_{L}}+\frac{C_{e}}{Q_{m}}$$

Freundlich:

(6) $$lnQ_{e}=\frac{1}{n}lnC_{e}+lnK_{F}$$

where *Q_e_* and *Q_m_* represent the adsorption capacity and the maximum adsorption capacity, respectively; *C_e_* is the concentration of pollutants in the solution at adsorption equilibrium; *K_L_* is the Langmuir adsorption equilibrium constant; and *K_F_* is the Freundlich constants with the affinity coefficient.

## 3. Result and Discussion

### 3.1. Performance Testing

In order to study the physical and chemical properties of the materials, the materials were screened by the adsorption catalytic performance of removing pollutant MB (Figure S1). It can be clearly seen from the figure that the adsorption–catalytic efficiency of several different materials for removing MB under the dark reaction and visible light conditions was B-TiO_2_/C_3_N_4_ > B-C_3_N_4_ > B2-C_3_N_4_/TiO_2_ > TiO_2_/C_3_N_4_ > B-TiO_2_ > g-C_3_N_4_ > TiO_2_. Among them, the adsorption and removal rate of MB on B-TiO_2_/C_3_N_4_ in the dark reaction process reached 73.8%, while that of B2-C_3_N_4_/TiO_2_ was only 17.8%. Then, under the visible light condition for 2 h, the removal rate of MB by B-TiO_2_/C_3_N_4_ reached 97.3%, while that of B2-C_3_N_4_/TiO_2_ only reached 66.5%. Therefore, according to the preliminary experimental results, the B-TiO_2_/C_3_N_4_ material was selected as the composite photocatalyst with the highest removal efficiency for follow-up research.

The mixing ratio and calcination temperature of the composites are both important factors affecting the performance of the composite materials. Therefore, in order to further analyze the performance of the composite material, this experiment first selected B_2_O_3_ and TiO_2_/C_3_N_4_ with different mass ratios to be mixed and calcined at 550 °C and then selected the material with the best pollutant removal effect for subsequent experiments.

According to the amount of doping of B_2_O_3_, the composite materials were marked as B-TiO_2_/C_3_N_4_-x (x = 1, 2, 3, 4, 5). The removal efficiencies are shown in Figure S2. The removal rates of B-TiO_2_/C_3_N_4_-1, B-TiO_2_/C_3_N_4_-2, and B-TiO_2_/C_3_N_4_-3 were 69.3%, 83.2%, and 73.4%, respectively, while the removal rates of B-TiO_2_/C_3_N_4_-4 and B-TiO_2_/C_3_N_4_-5 were only 50.9% and 23.9%.

Therefore, according to the above experimental results, B-TiO_2_/C_3_N_4_-2 had the best effect and was selected as the follow-up research material. A 2 g amount of B_2_O_3_ and 1 g of TiO_2_/C_3_N_4_ material were mixed uniformly, and the mixture temperature was heated at a rate of 5 °C/min for 2 h at different temperatures. The set target temperatures were 350 °C, 450 °C, 550 °C, 650 °C, and 750 °C. For convenience of description, B-TiO_2_/C_3_N_4_ prepared at different calcination temperatures was named after its temperature (350 °C, 450 °C, 550 °C, 650 °C, and 750 °C).

### 3.2. Characterization and Analysis of B-TiO_2_/C_3_N_4_ Composites

**Figure 1:**
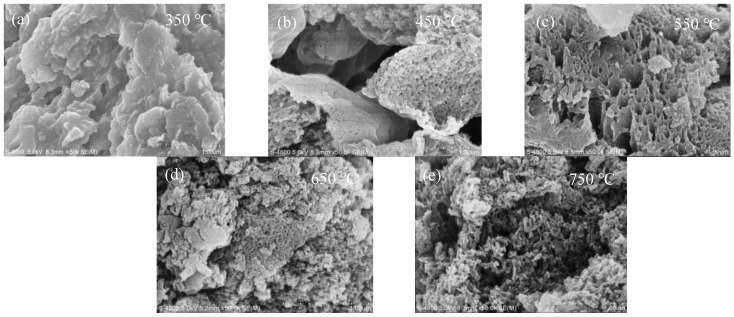
SEM of B-TiO_2_/C_3_N_4_ under different calcination conditions: (**a**) 350 °C; (**b**) 450 °C; (**c**) 550 °C; (**d**) 650 °C; (**e**) 750 °C.

The morphology of B-TiO_2_/C_3_N_4_ composites prepared under different calcination conditions at different temperatures are shown in Figure 1. It can be seen from Figure 1a that at 350 °C the surface of the structure was relatively smooth, which may have been due to molten B_2_O_3_ wrapping the base material. Figure 1b shows the surface layer of the calcined material at 450 °C was still partially covered, but a large number of loose structures also appeared. This may have been due to the increase in the calcination temperature, and the disorder degree increased due to the B_2_O_3_ entering the molten state, which contains polar -B=O groups. As seen in Figure 1c, many loose pores appeared on the surface of the composite under the 550 °C calcination condition. As the temperature continued to increase, the main structure of the g-C_3_N_4_ material began to decompose when the temperature was above 600 °C, and the pulverization of the g-C_3_N_4_ structure can be clearly seen in Figure 1d. At 750 °C, g-C_3_N_4_ was decomposed into nitrogen and nitrile-based fragments, and TiO_2_ was gradually transformed from anatase to rutile; so, the layered structure of g-C_3_N_4_ cannot be observed in Figure 1e, while some agglomerates of particles can be seen.

The specific surface area and porosity of materials are crucial factors affecting the adsorption and photocatalytic reactions. As shown in Figure S3, the representative N_2_ adsorption–desorption isotherms of composite materials were of type IV [29]. According to the classification of IUPAC, the adsorption and desorption curve hysteresis loop types of the prepared B-TiO_2_/C_3_N_4_ materials belonged to the “H3” hysteresis loop (0.5 < P/P_0_ < 0.98:550 °C, 650 °C, 750 °C; 0.75 < P/P_0_ < 0.97:350 °C, 450 °C). The low-temperature calcination shown for loops located at high P/P_0_ was caused by the aggregation of the 2D lamellar structure of g-C_3_N_4_. By contrast, the high temperature and B_2_O_3_ as a medium effectively resulted in more holes on the surface of g-C_3_N_4_. In Table 1, details regarding the specific surface areas, average pore diameters, and pore volumes of various materials are summarized. Among the temperature series, 550 °C showed the highest specific surface areas, average pore diameters, and pore volumes. This is because, at the calcination temperature of 550 °C, the molten B_2_O_3_ could fully contact the TiO_2_/C_3_N_4_ composite as a reaction environment modifier. The lamellar structure of g-C_3_N_4_ was exfoliated in the molten environment while the pore structure was formed during the cooling process, so the specific surface area was significantly increased.

**Figure 2:**
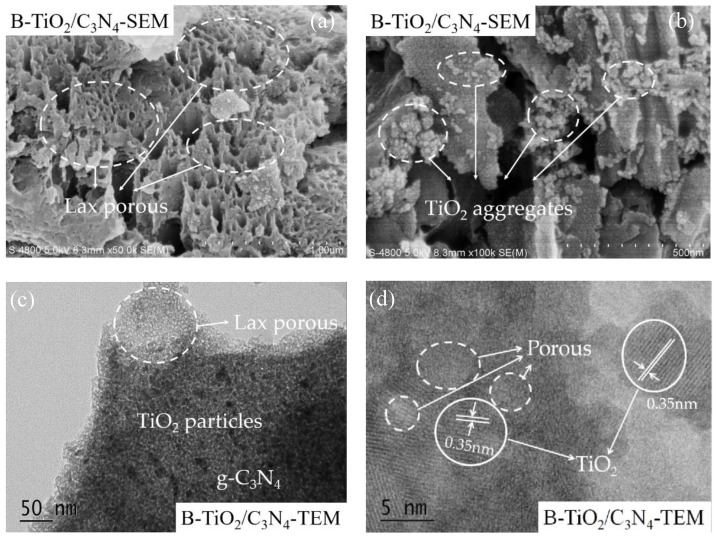
SEM (**a**,**b**) and TEM (**c**,**d**) spectra of B-TiO_2_/C_3_N_4_ (550 °C) composite materials.

The surface morphologies and microstructure of B-TiO_2_/C_3_N_4_ (550 °C) composite material are shown in Figure 2. From Figure 2a,b, it can be clearly observed that there were a lot of loose macropores and particle agglomeration on the surface of the g-C_3_N_4_-based material. In order to better analyze the morphological characteristics of the material, the high-resolution transmission electron microscope (HR-TEM) pattern was used for analysis. In Figure 2c, it can be seen that there were a lot of loose pores on the surface of the g-C_3_N_4_. Otherwise, a large number of granular material lattice fringes appeared on the g-C_3_N_4_ lamellar structure. The lattice spacing embedded particles were measured to be ~0.35 nm (Figure 2d), which is similar to the TiO_2_ (101) crystal plane structure [30]. Through the analysis of specific surface area and the characterization of SEM and TEM, it was found that TiO_2_ grew uniformly on the surface of the g-C_3_N_4_ layered structure, and the modification of the TiO_2_/C_3_N_4_ composite structure by B_2_O_3_ produced a large number of loose pores on the g-C_3_N_4_ layered structure. Such a modification method not only improved the specific surface area of the material, but also preserved the composite structure of TiO_2_/C_3_N_4_, which is more conducive to the charge transfer of photogenerated carriers between composite semiconductor materials and improves the photocatalytic activity of the material.

The XRD pattern of materials was recorded and is shown in Figure S4. It can be seen that B_2_O_3_ showed two obvious, characteristic peaks at 14.6°and 27.9° [31]. The composites prepared at 350 °C and 450 °C had only two obvious characteristic peaks of B_2_O_3_ but no characteristic peaks of TiO_2_ and g-C_3_N_4_ [32]. This may have been because the surface of the TiO_2_/C_3_N_4_ material was coated by B_2_O_3_ in the molten state at 350 °C and 450 °C, which is basically consistent with the SEM characterization shown in Figure 1. At the same time, due to the large amount of B_2_O_3_ added, there was no peak position of g-C_3_N_4_ and TiO_2_ but only the characteristic peak of B_2_O_3_. At 550 °C, the two characteristic peaks of B_2_O_3_ disappeared in the XRD pattern, and the anatase (25.3°) and rutile (27.4°) diffraction peaks of TiO_2_ appeared. It was caused by the entry of polar -B=O into the TiO_2_/C_3_N_4_. When the temperature rose to 650 °C, several characteristic peaks (27.4°, 41.3°, etc.) of the rutile phase of TiO_2_ became more and more obvious, which indicated that the crystal structure of TiO_2_ gradually changed from anatase to rutile with the increase in temperature. It can be seen from XRD analysis that when the temperature gradually increased, the degree of disorder gradually increased, and a polar -B=O bond was formed to form a new structure with TiO_2_ or g-C_3_N_4_ [33].

The Fourier-transform infrared spectroscopy of composite materials is shown in Figure S5. In this figure, it can be seen that the peak at 550 °C was the smallest, which was the Ti–O bond expansion joint and Ti stretching vibration of the -O–Ti bond. Compared with the B-TiO_2_/C_3_N_4_ composites at different calcination temperatures (350 °C, 450 °C, 550 °C, 650 °C, and 750 °C), the obvious peaks appeared at 810 cm^−1^ and 1200–1600 cm^−1^. The characteristic peak at 810 cm^−1^ was the stretching vibration of the triazine ring structure in g-C_3_N_4_, while the broad peak spectrum at 1200–1600 cm^−1^ was caused by the stretching vibration of C=N. Otherwise, the obvious differences of all composite materials were mainly due to the incomplete transformation of B_2_O_3_ from solid to molten state. At low temperatures, the peaks were not obvious, which was caused by molten B_2_O_3_ coating on the surface of composite material, resulting in limited exposure of the surface structure of the B-TiO_2_/C_3_N_4_ composite. In addition, the absorption peaks of B-TiO_2_/C_3_N_4_ composites at 3000–3300 cm^−1^ were mainly the stretching vibration of the amino group (-NH_2_). Meanwhile, the two peaks at 2260 cm^−1^ and 2350 cm^−1^ were the C=O stretching vibration of CO_2_ in the atmosphere [34].

Figure S6 shows the XPS spectra of several elements of B-TiO_2_/C_3_N_4_: B1s, N1s, C1s, Ti2s and O1s. B1s shows three binding energies at 190.4 eV, 192.7 eV, and 193.5 eV, of which 192.7 eV belonged to the B–O bond, 190.4 eV was the binding energy of the B–N bond, and 193.5 eV may have been O–B formed by B entering the TiO_2_ crystal structure—Ti or TiB_2_ bond [35]. This indicates that B element entered into the structures of TiO_2_ and g-C_3_N_4_. C1s showed three main binding energies, of which 284.8 eV was the typical sp2 hybridization of the graphite phase (C=C bond peak position), and 288.7 eV corresponded to the C–N–C bond in the structure. In addition, 286.5 eV corresponded to the exocyclic C–O of carbon–nitrogen polymer materials [36]. Figure S6c shows the N1s binding energy peaks at 397.3 eV, 398.2 eV, and 401.9 eV. Among them, 398.2 eV was attributed to the sp2 hybridization (C–N=C) of the N triazine ring structure, while the peak at 401.9 eV corresponded to the bridged N atom (N–(C)^3^) [37], and the peak at 397.3 eV corresponded to N–B [38]. In addition, the characteristic peaks in the N spectrum were slightly shifted, possibly due to the increased delocalization between the large π bonds in the carbon nitride structure due to the doping of B element [29]. The two peaks of 458.7 eV and 464.8 eV in the binding energy of Ti 2s were the electron binding energies of Ti^4+^ 2p^3/2^ and 2p^1/2^ in TiO_2_, respectively. The strong peak at 530.1 eV in the O1s spectrum was the O–Ti bond in TiO_2_, while 531.0 eV and 532.7 eV were the surface-adsorbed water molecules, H_2_O and surface hydroxyl groups (-OH), respectively [39].

Figure S7 shows the UV–Vis diffuse reflectance absorption spectra of the composite material (350 °C, 450 °C, 550 °C, 650 °C, 750 °C). It can be seen from the figure that under different temperature conditions, the UV–visible light absorption properties of the composites were different. The B-TiO_2_/C_3_N_4_ prepared at the three temperatures of 350 °C, 450 °C, and 550 °C had better visible light absorption ability in both the ultraviolet and visible light regions, and the effect of 550 °C was the best. This result was caused by B element entering into the TiO_2_ lattice and g-C_3_N_4_ structure. According to the low electronegativity of B element, it can be inferred that the 2p orbital of B may be different from the 2p orbital of O. Hybridization was generated, thus, enabling enhanced visible light absorption [40]. Otherwise, the 650 °C and 750 °C had high light absorption capacity in the ultraviolet regions of 200–350 nm and 200–400 nm, respectively. The light absorption ability of the visible light region greater than 400 nm was weak. This may have been due to the gradual decomposition of the g-C_3_N_4_ structure in the composite material with the increase in temperature, resulting in a decrease in the visible light absorption capacity of the material. At the same time, although the temperature increased, it could also have caused the transformation of the TiO_2_ crystal phase, while the mixed-phase TiO_2_ had visible light absorption ability, but the effect was not obvious. Therefore, according to the UV–Vis diffuse reflectance spectrum analysis, it is shown that the B-TiO_2_/C_3_N_4_ composites prepared at 550 °C had good light absorption ability.

Based on the above characterization analysis description, we can roughly infer the preparation process of composite materials. The TiO_2_/C_3_N_4_ material was exfoliated by using the molten reaction environment provided by B_2_O_3_ during the heating process. At the same time, the gas was decomposed by the trace amount of g-C_3_N_4_, thereby forming a large number of loose pore structures. With the increase in calcination temperature, the generated polar -B=O bond replaced the H atom of the amino group on carbon nitride melon and formed an O–B–Ti or TiB_2_ structure, thereby finally forming a structurally stable B-TiO_2_/C_3_N_4_ material. The specific reaction process inference diagram is shown in Figure S8.

### 3.3. Photocatalytic Efficiencies

**Figure 3:**
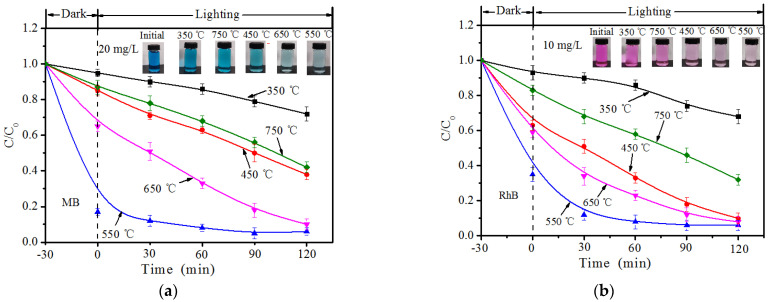
The adsorption–photocatalytic degradation curves of MB (**a**) and RhB (**b**) over B-TiO_2_/C_3_N_4_.

Figure 3 shows the dark adsorption and light-driven photocatalytic degradation ability of MB (a) and RhB (b) of B-TiO_2_/C_3_N_4_ composite materials (350 °C, 450 °C, 550 °C, 650 °C, and 750 °C). The specific experimental process was as follows: 30 mg of B-TiO_2_/C_3_N_4_ composite was added into 100 mL of MB solution with a concentration of 20 mg/L and 10 mg/L of RhB solution, respectively. A half-hour dark reaction time was set in the experiment and then the photocatalytic degradation experiment was carried out. The removal ability of RhB and MB was analyzed by catalytically degrading RhB and MB under the irradiation of a xenon light source with a wavelength greater than 400 nm.

**Figure 4:**
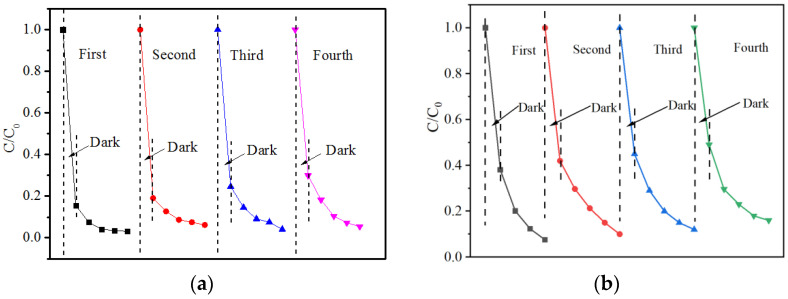
Cycling experiments of B-TiO_2_/C_3_N_4_ for the adsorption–photocatalytic degradation of MB (**a**) and RhB (**b**).

The calculation of the removal efficiency of each pollutant by several catalyst materials was calculated by Formula (1). The experimental results of the dark reaction and the light reaction are shown in Figure 4a,b. The two figures show the adsorption–degradation curves of MB (20 mg/L) and RhB (10 mg/L) by the B-TiO_2_/C_3_N_4_ composite, respectively. The relationship among the adsorption–degradation efficiencies can be clearly seen from the figure: 550 °C > 650 °C > 450 °C > 750 °C > 350 °C. The B-TiO_2_/C_3_N_4_ prepared at the 550 °C calcination temperature had the best adsorption effect on the two dyes in the dark reaction adsorption process and the best removal efficiency (83.4% (MB), 64.1% (RhB)). At this temperature, B_2_O_3_ may undergo a complete molten state process, and become a good molten state reaction environment to fully contact with TiO_2_/C_3_N_4_. Therefore, the prepared B-TiO_2_/C_3_N_4_ had a larger specific surface area, exposing more adsorption sites and a reaction active site. Therefore, the doping of B element is beneficial to improve photocatalytic activity. When the temperature continued to rise above 600 °C, the g-C_3_N_4_ material began to decompose gradually, and the TiO_2_ gradually transformed from anatase type to rutile phase with lower photocatalytic reaction activity, so the photocatalytic reaction efficiency decreased gradually.

Meanwhile, multiple cycle experiments were carried out with MB and RhB. It can be seen from Figure 4 that after four cycles of repeated tests, the adsorption and removal rate of B-TiO_2_/C_3_N_4_ to 20 mg/L organic solution decreased from 83.4% to 70.1%(MB) and from 64.1% to 51.2% (RhB). The results showed that the adsorption effect of the material on MB and RhB was weakened gradually. This may have been because the pores on the surface of the material were damaged during the experiment. Meanwhile, the active sites were reduced due to the coverage of undegraded pollutants adsorbed on the surface of the material, resulting in a decrease in the adsorption and catalytic efficiency of the material after multiple experiments. Although the adsorption capacity of the material for the two pollutions was gradually weakened, the removal rate remained at around 95% (MB) and 80% (RhB) within two hours. This result shows that the B-TiO_2_/C_3_N_4_ composite material had better stability and repeatability.

The TOC in the photocatalytic degradation system reflected the mineralization degree of organic pollutants. The results of the TOC are shown in Figure S9. The adsorption process effectively reduced the value. With the progress of visible light, the TOC value experienced small decreases with the prolongation of the lighting time. This is because, during the degradation process, the pollutant molecules were decomposed from macromolecules to small molecules containing organic carbon, and the pollutants adsorbed on the surface of the material were also desorbed. The resorption–degradation process resulted in insignificant changes in the organic carbon content in the solution [41,42].

### 3.4. Adsorption Property

To demonstrate the adsorption property of B-TiO_2_/C_3_N_4_ (550 °C) composite materials, adsorption and removal rates of RhB and MB were chosen as the contaminant. The adsorption efficiency plays an important role in the rapid removal of pollutants. 

**Figure 5:**
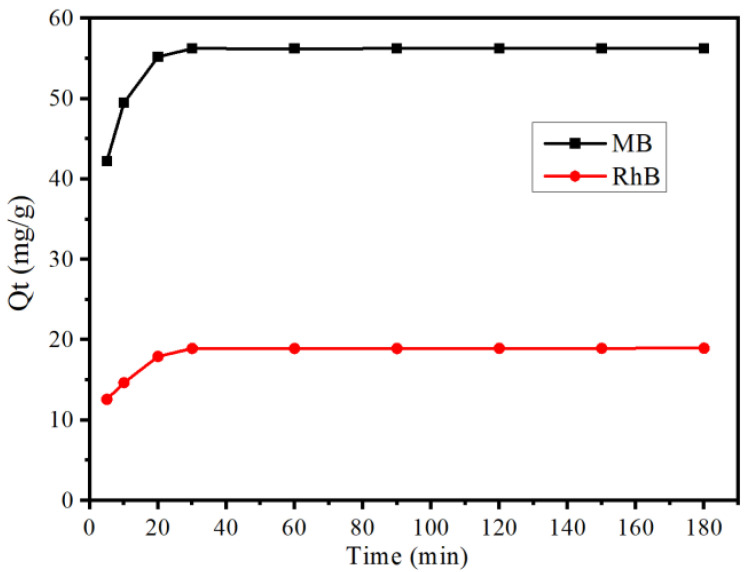
Adsorption kinetics curves of B-TiO_2_/C_4_ on MB and RhB.

The kinetic curve of adsorption of MB and RhB on B-TiO_2_/C_3_N_4_ is shown in Figure 5. From this figure, it can be seen that B-TiO_2_/C_3_N_4_ reached the adsorption equilibrium for the two pollutants within 30 min; the adsorption capacity of MB reached 56.25 mg/g; and the adsorption capacity of RhB reached 18.94 mg/g. Compared with other materials (as shown in Figure S1), the improvement of adsorption efficiency was due to the increase in specific surface area and the increase in adsorption sites, and the doping of B element in g-C_3_N_4_ expanded the large, π-bonded conjugated system, which was more conducive to the adsorption of MB [9].

**Figure 6:**
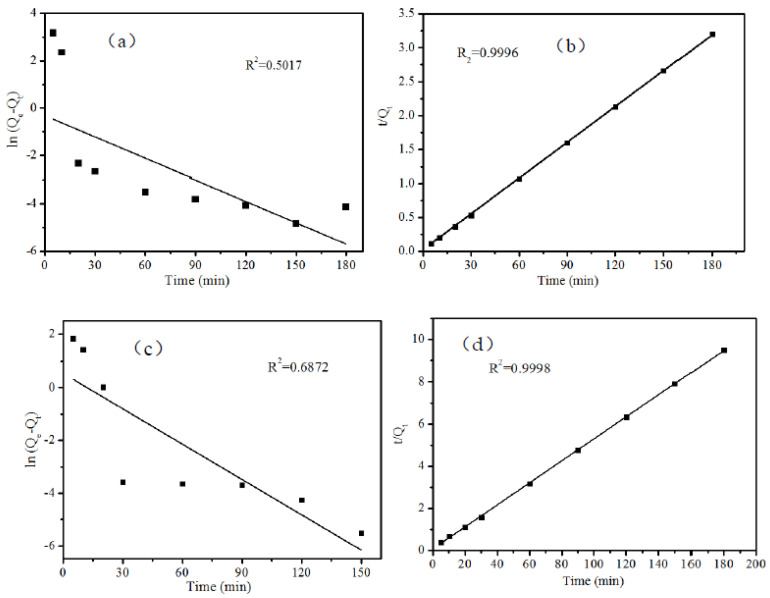
(**a**) Plots of the pseudo-first-order model (MB); (**b**) Plots of the pseudo-second-order model (MB); (**c**) Plots of the pseudo-first-order model (RhB); (**d**) Plots of the pseudo-second-order model (RhB).

The pseudo-first-order kinetic and pseudo-second-order kinetic models were fitted to the adsorption amounts of MB and RhB, respectively. The results are shown in Figure 6. The fitted correlation coefficients were: MB: R_1_^2^ = 0.5017 (a), R_2_^2^ = 0.9964 (b); RhB: R_1_^2^ = 0.6872 (c), R_2_^2^ = 0.9998 (d). According to the correlation coefficients fitted to the two pollutants, it can be seen that the pseudo-second-order kinetic model better fitted the adsorption behavior of B-TiO_2_/C_3_N_4_.

**Figure 7:**
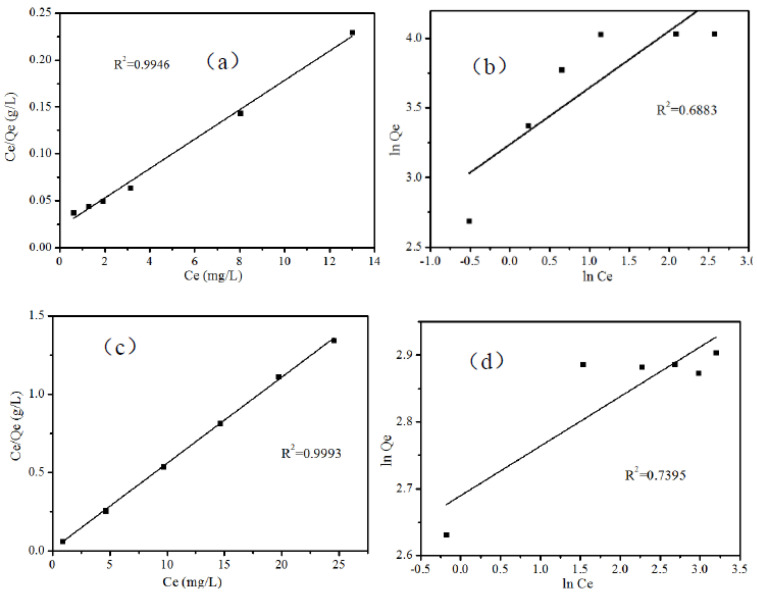
The linearized of Langmuir adsorption isotherm (**a**) MB (**c**) RhB and Freundlich adsorption isotherm (**b**) MB (**d**) RhB adsorption by B-TiO_2_/C_3_N_4_.

Figure 7 shows the fitting of the Langmuir and Freundlich adsorption isotherm models for the adsorption of MB (a) (b) and RhB (c) (d) on B-TiO_2_/C_3_N_4_, respectively. By comparing the correlation coefficients obtained by calculation, it was found that for the MB adsorption isotherm model, R_L_^2^ = 0.9946 was higher than that of the Freundlich model (R_F_^2^ = 0.6883), while the simulated adsorption isotherm model for RhB had the same or similar results (R_L_^2^: 0.9993 > R_F_^2^: 0.7395). This result showed that the adsorption of MB and RhB by B-TiO_2_/C_3_N_4_ was monolayer adsorption, and the adsorption was more favorable with the increase in pollutant concentration. Therefore, the adsorption of these two pollutants by B-TiO_2_/C_3_N_4_ was more inclined to the Langmuir adsorption isotherm model.

### 3.5. Analysis of Photocatalytic Mechanism

**Figure 8:**
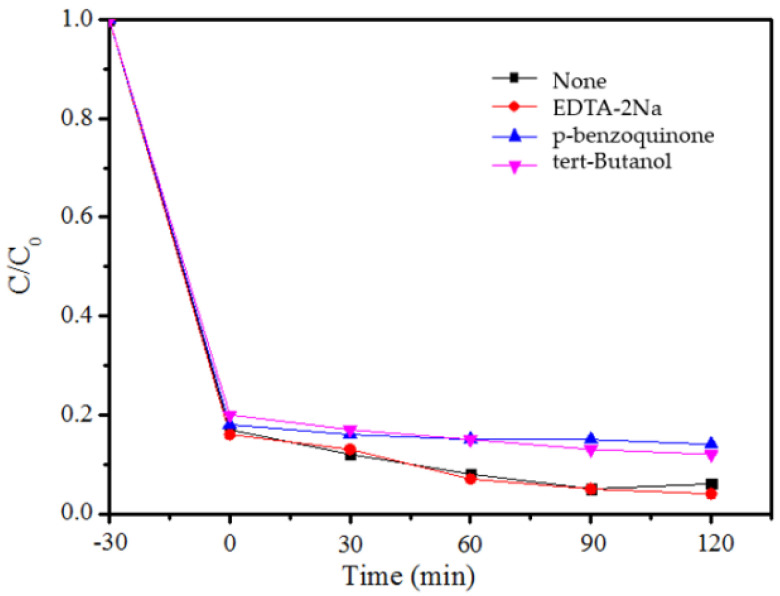
The capture experiments of MB by B-TiO_2_/C_3_N_4_.

Based on the above experimental results, MB was selected as the target pollutant with the best removal effect, and the main substances involved in the photocatalytic reaction were determined by the free radical capture experiment. The experimental results are shown in Figure 8. The addition of EDTA-2Na had less of an effect on the degradation effect of MB, which indicates that the role of holes is less important in the catalytic reaction. After the addition of p-benzoquinone, the degradation of MB was inhibited, indicating that superoxide radical (∙O^2−^) is an important free radical involved in the photodegradation process. After the addition of tert-Butanol, the degradation of MB was also inhibited to a certain extent, so it can be concluded that hydroxyl radicals (∙OH) are also involved in the photocatalytic oxidation process. The above research results show that ∙O^2−^ and ∙OH radicals were the active groups that mainly participated in the reaction during the degradation of MB by B-TiO_2_/C_3_N_4_.

**Figure 9:**
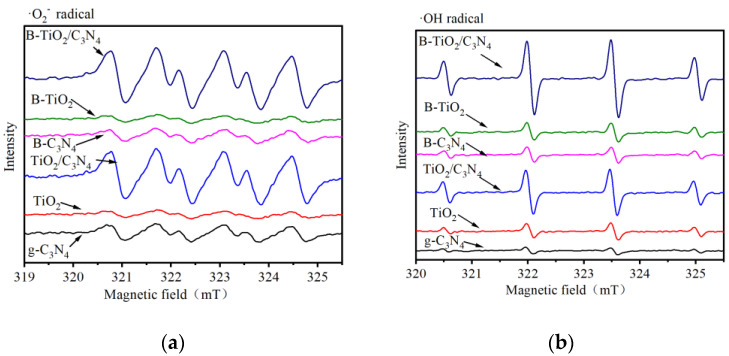
Spectrum of DMPO spin trapping ESR of different materials (**a**) DMPO-O_2_^−^ and (**b**) DMPO-·OH.

Through mechanism analysis, it was found that ∙O^2−^ and ∙OH were the main active groups involved in the photocatalytic reaction. Therefore, DMPO ESR was used to detect the ∙O^2−^ and ∙OH produced by B-TiO_2_/C_3_N_4_ material under visible light above 400 nm. In addition, a comprehensive comparative analysis was conducted with other materials. It can be clearly seen from Figure 9a that after 10 min of light source irradiation, the order of ∙O^2−^ESR signal intensity was B-TiO_2_/C_3_N_4_ > TiO_2_/C_3_N_4_ > g-C_3_N_4_ > B-C_3_N_4_ > B-TiO_2_ > TiO_2_. The signal peak generated by B-TiO_2_/C_3_N_4_ was slightly higher than that of TiO_2_/C_3_N_4_ and significantly higher than that of the other three materials. In the ESR spectrum of ∙Oh, as shown in Figure 9b, the signal peak generated by B-TiO_2_/C_3_N_4_ was the strongest. According to the analysis results, the B-TiO_2_/C_3_N_4_ material produced the most ∙O^2−^ and ∙OH in the visible light photocatalytic system, so it had a better photocatalytic effect.

**Figure 10:**
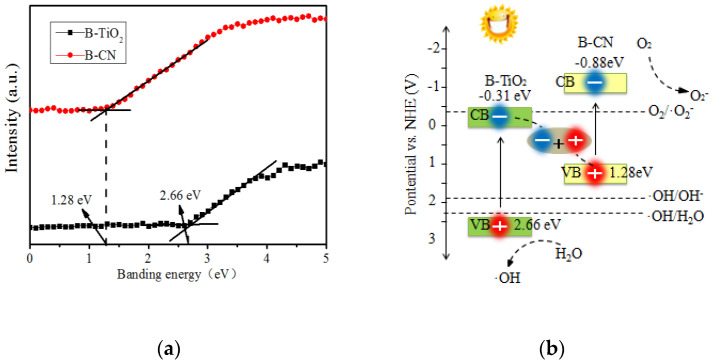
The photocatalytic reaction mechanism of B-TiO_2_/C_3_N_4_: (**a**) The VBXPS of B-TiO_2_ and B-CN; (**b**) Scheme of proposed mechanism for degradation of B-TiO_2_/C_3_N_4_.

Otherwise, the XPS characterization showed that the B element in B_2_O_3_ entered the TiO_2_ crystal structure. Therefore, in order to analyze the structural composition of B-TiO_2_ and B-C_3_N_4_, the conduction band relationship of the forbidden band width of B-TiO_2_ and B-C_3_N_4_ was analyzed. The specific results are shown in Figure 10a.

The photocatalytic reaction mechanism of B-TiO_2_/C_3_N_4_ composite material was deduced according to the radical trapping experiment. As shown in Figure 10b, under visible light conditions, the conduction band position of B-C_3_N_4_ was −0.88 eV, which was more negative than that of B-TiO_2_ (−0.31 eV) and E0 (O_2_/·O^2−^ = −0.33 eV). The electrons could be captured by O_2_ to form ·O^2−^ and finally formed H_2_O_2_ on the surface of the material. The holes were trapped by H_2_O and OH^−^ in the valence band of B-TiO_2_ to form hydroxyl radicals (·OH). These species are vital for photocatalytic degradation of organic pollution. Meanwhile, it can be determined that the B-TiO_2_/C_3_N_4_ composite material formed a Z-type heterostructure [43,44].

## 4. Conclusions

Herein, B-TiO_2_/C_3_N_4_ composites were prepared by modifying Z-type heterostructured TiO_2_/C_3_N_4_ composites using the molten reaction environment created by B_2_O_3_. The physical-chemical properties of B-TiO_2_/C_3_N_4_ composites prepared at different temperatures were analyzed by various characterization methods. Through characterization, it was found that the addition of B_2_O_3_ effectively increased the specific surface area of TiO_2_/C_3_N_4_ materials, thereby increasing the active sites for adsorption and photocatalysis. Crystal analysis showed that the modification of TiO_2_/C_3_N_4_ by B_2_O_3_ simultaneously doped B element into the TiO_2_ lattice and g-C_3_N_4_, which helped to inhibit the recombination of electron holes and improved the photocatalytic activity. At the same time, the doping of B element expanded the π-conjugated system of the g-C_3_N_4_ material, which is beneficial for the improvement of MB adsorption performance. Above all, this paper started with consideration of the two aspects of improving the adsorption capacity and photocatalytic oxidation capacity of the material and integrated the preparation, characterization, and adsorption–degradation mechanism analysis of the material, providing theoretical support and experimental basis for research on the synergistic removal of pollutants by adsorption and photocatalysis.

## Figures and Tables

**Table 1:** BET surface area, average pore size, and pore volume of B-TiO_2_/C_3_N_4_ material at different temperatures.

Sample	350 °C	450 °C	550 °C	650 °C	750 °C
BET (m^2^/g)	13.381	13.472	58.654	34.622	25.795
Average pore size (nm)	6.311	7.809	6.960	12.733	14.263
Average pore volume (cm^3^/g)	0.090	0.073	0.130	0.162	0.103

## Data Availability

Not applicable.

## Supplementary Materials

The following supporting information can be downloaded at: https://www.mdpi.com/article/10.3390/ijerph19148683/s1, Table S1: Comparison of MB removal rate of different materials, Figure S1: MB removal efficiency of several materials: TiO_2_, g-C_3_N_4_, B-TiO_2_, B-C_3_N_4_, TiO_2_/C_3_N_4_, B2-C_3_N_4_/TiO_2_ and B-TiO_2_/C_3_N_4_, Figure S2: MB removal efficiency of B-TiO_2_/C_3_N_4_ prepared with different doping amount, Figure S3: N_2_ adsorption-desorption curves of B-TiO_2_/C_3_N_4_ materials at different temperatures, Figure S4: XRD (X-ray diffraction) patterns of B_2_O_3_ and B-TiO_2_/C_3_N_4_-2 Series materials, Figure S5: FT-IR spectra for different temperature B-TiO_2_/C_3_N_4_, Figure S6: XPS spectra of B-TiO_2_/C_3_N_4_ material (a)B1s, (b)C1s, (c) N1s, (d) Ti2p and (e) O1s, Figure S7. The Diffuse reflectance spectra for different temperature B-TiO_2_/C_3_N_4_, Figure S8. Synthesis path of B-TiO_2_/C_3_N_4_ material, Figure S9. Changes of TOC in the experiment of adsorption-catalytic degradation of pollutants (a) MB (b) RhB. References [45,46,47] are cited in the supplementary materials.
