# Supplementary material for: Preparation of High-Porosity B-TiO2/C3N4 Composite Materials: Adsorption–Degradation Capacity and Photo-Regeneration Properties
Source: Int J Environ Res Public Health. 2022 Jul 17;19(14):8683. doi: 10.3390/ijerph19148683 (PMC9319032; doi:10.3390/ijerph19148683)
Supplement: Supplementary file 1 [file ijerph-19-08683-s001.zip › ijerph-1808776-supplementary.pdf]

# Supplementary Information

# Preparation of High-Porosity B-$TiO_2$/$C_3N_4$ Composite Materials: Adsorption–Degradation Capacity and Photo-Regeneration Properties

Xiang Guo[1], Lei Rao[2,*] and Zhenyu Shi[1]

1 College of Environment, Hohai University, Nanjing 210098, China; gx2015@hhu.edu.cn

2 College of Mechanics and Materials, Hohai University, Nanjing 21100, China; raol@hhu.edu.cn

* Correspondence: raol@hhu.edu.cn;

## Supplementary Text

The composite B-$TiO_2$/$C_3N_4$ of this work was compared previously reported under similar conditions (Table 1). From the above comparison and the performed tests, it can be concluded that the MB adsorption and photocatalytic rate of B-$TiO_2$/$C_3N_4$ is enhanced clearly.

**Table S1.** Comparison of MB removal rate of different materials

| Catalysts | Mass of catalyst | Experimental conditions | Removal efficiency | Reference |
|---|---|---|---|---|
| **B-$TiO_2$/$C_3N_4$** | 30 mg | 100ml of 20 mg/L MB | 73.8%（Dark reaction in 30 min）<br>97.3%（Light reaction in 2h） | This work |
| **B-$C_3N_4$** | 30 mg | 100ml of 20 mg/L MB | 43.11%（Dark reaction in 30 min）96.8% ( Light reaction in 2h) | [1] |
| **H-g-$C_3N_4$** | 0.1g | 100ml of 1000 mg/L MB | 43.23%（Dark reaction in 60 min）<br>96.61% ( Light reaction in 2h) | [2] |
| **Ag-ZnO** | 0.1% | 20ppm | 67.3% (Light reaction in 3.5h) | [3] |
| **$TiO_2$** | 30mg | 100ml of 20 mg/L MB | 4.1%（Dark reaction in 30 min）<br>19.8%（Light reaction in 2h） | This work |
| **g- $C_3N_4$(650℃)** | 25mg | 50ml of 10 mg/L MB | 34.5%（Dark reaction in 60 min）<br>81.2%（Light reaction in 3h） | [4] |

## References

*1. Guo, X.; Rao, L.; Wang, P.; Zhang, L.; Wang, Y. Synthesis of Porous Boron-Doped Carbon Nitride: Adsorption Capacity and Photo-Regeneration Properties. International Journal of Environmental Research and Public Health 2019, 16, 581, doi:10.3390/ijerph16040581.*

*2. Feng, J.; Chen, T.; Liu, S.; Zhou, Q.; Ren, Y.; Lv, Y.; Fan, Z. Improvement of g-$C_3N_4$ Photocatalytic Properties Using the Hummers Method. Journal of Colloid and Interface Science 2016, 479, 1–6, doi:10.1016/j.jcis.2016.06.040.*

*3. Kwon, D.; Kim, J. Silver-Doped ZnO for Photocatalytic Degradation of Methylene Blue. Korean J. Chem. Eng. 2020, 37, 1226–1232, doi:10.1007/s11814-020-0520-7.*

4. Mo, Z.; She, X.; Li, Y.; Liu, L.; Huang, L.; Chen, Z.; Zhang, Q.; Xu, H.; Li, H. Synthesis of g-$C_3N_4$ at Different Temperatures for Superior Visible/UV Photocatalytic Performance and Photoelectrochemical Sensing of MB Solution. RSC Adv. 2015, 5, 101552–101562, doi:10.1039/C5RA19586A.

# Supplementary Figures

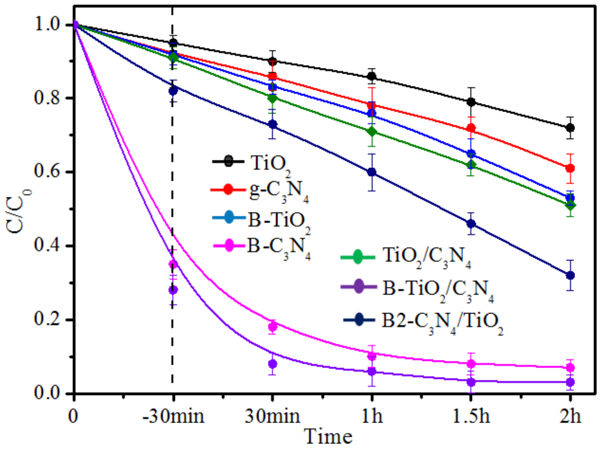


**Figure S1.** MB removal efficiency of several materials: $TiO_2$, g-$C_3N_4$, B-$TiO_2$, B- $C_3N_4$, $TiO_2/C_3N_4$, B2-$C_3N_4/TiO_2$ and B-$TiO_2/C_3N_4$.

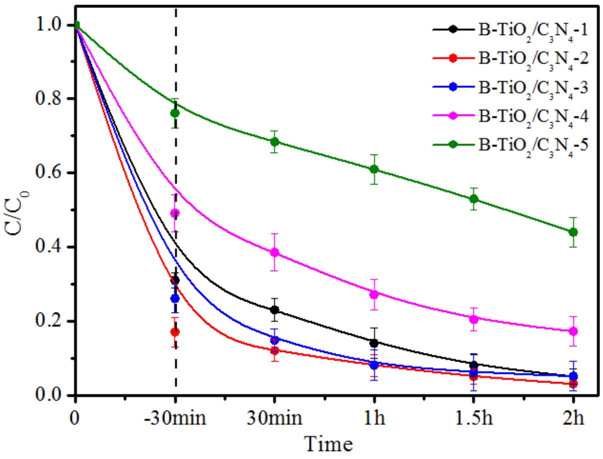


**Figure S2.** MB removal efficiency of B-$TiO_2/C_3N_4$ prepared with different doping amount.

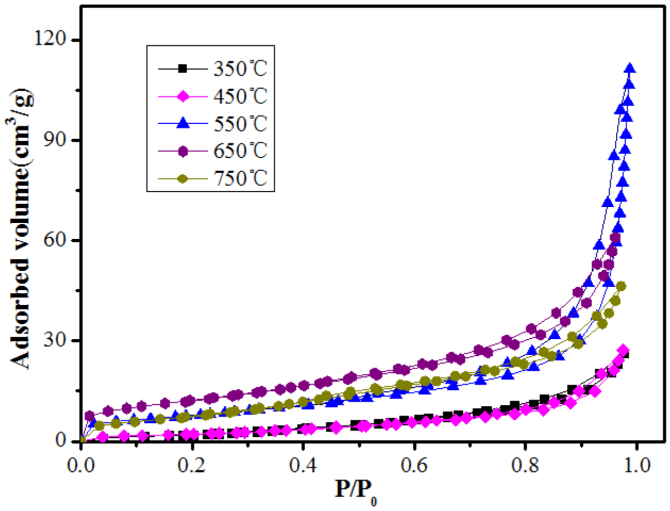


**Figure S3.** $N_2$ adsorption-desorption curves of B-$TiO_2$/$C_3N_4$ materials at different temperatures.

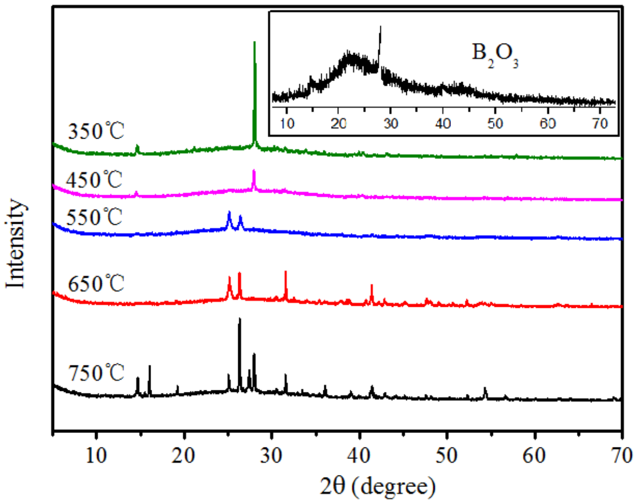


**Figure S4.** XRD (X-ray diffraction) patterns of $B_2O_3$ and B-$TiO_2$/$C_3N_4$-2 Series materials.

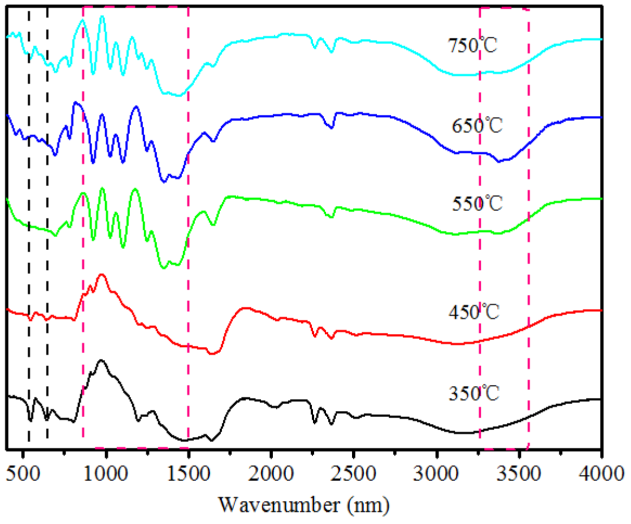


**Figure S5.** FT-IR spectra for different temperature B-$TiO_2$/$C_3N_4$.

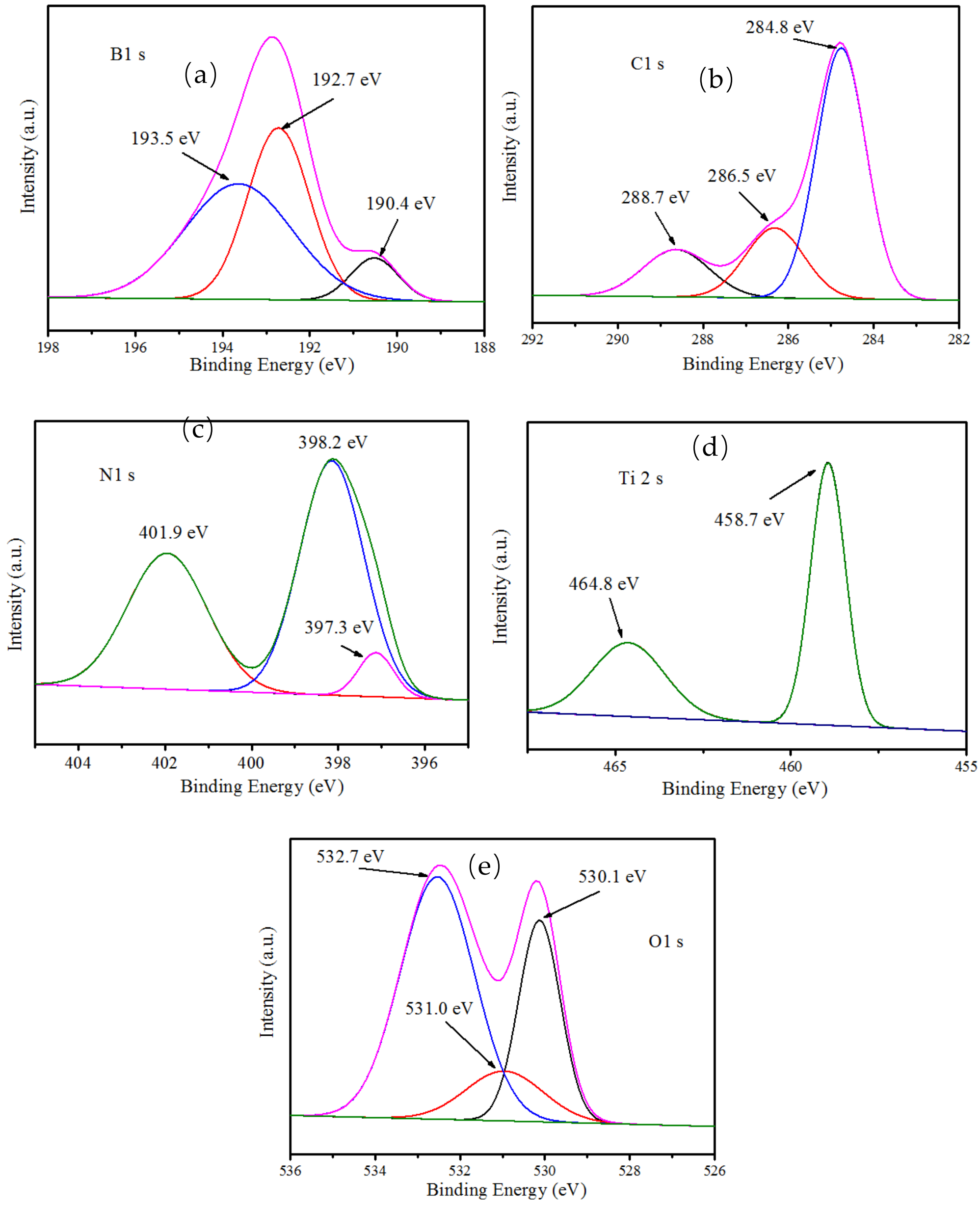


**Figure S6.** XPS spectra of B-$TiO_2$/$C_3N_4$ material (a)B1s, (b)C1s,(c) N1s,(d) Ti2p and (e) O1s.

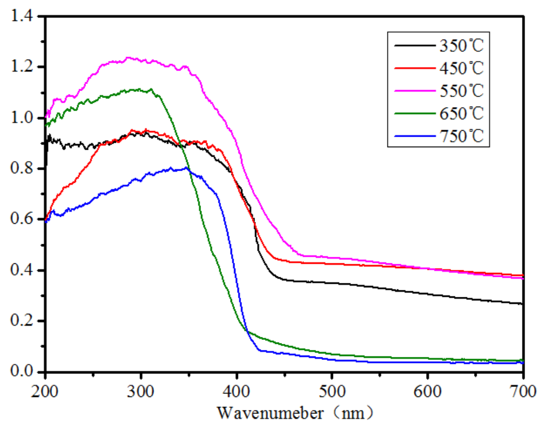


**Figure S7.** The Diffuse reflectance spectra for different temperature B-$TiO_2$/$C_3N_4$

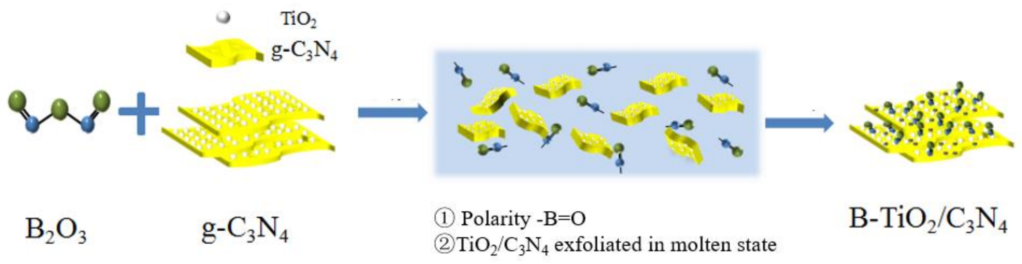


**Figure S8.** Synthesis path of B-$TiO_2$/$C_3N_4$ material

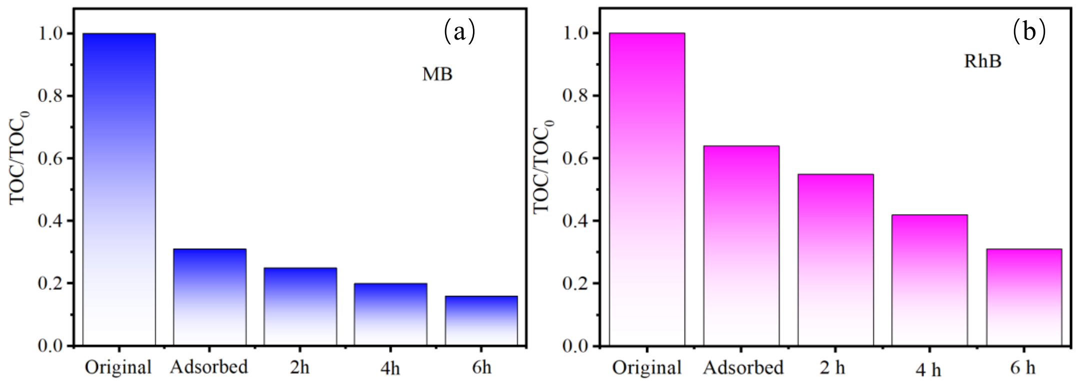


**Figure S9.** Changes of TOC in the experiment of adsorption-catalytic degradation of pollutants (a) MB (b) RhB
